# Hemoglobin Sunshine Seth: A Case Report of Low-Oxygen-Affinity Hemoglobinopathy

**DOI:** 10.1155/2020/2853531

**Published:** 2020-02-10

**Authors:** Leah S. Heidenreich, Jennifer L. Oliveira, Peter J. Holmberg, Vilmarie Rodriguez

**Affiliations:** ^1^Mayo Clinic Children's Center, Mayo Clinic, Rochester, Minnesota, USA; ^2^Division of Hematopathology, Mayo Clinic, Rochester, Minnesota, USA; ^3^Division of General Pediatric and Adolescent Medicine, Mayo Clinic, Rochester, Minnesota, USA; ^4^Division of Pediatric Hematology/Oncology, Mayo Clinic, Rochester, Minnesota, USA

## Abstract

Pulse oximetry is routinely used in the newborn nursery for clinical monitoring and to detect critical congenital heart disease. The differential diagnoses for reduced peripheral oxygen saturation in an infant include congenital heart disease, respiratory distress syndrome, transient tachypnea of the newborn, persistent pulmonary hypertension of the newborn, meconium aspiration syndrome, pneumonia, pneumothorax, and sepsis. The diagnostic evaluation for neonatal hypoxemia can be invasive and expensive. When this evaluation is unrevealing, other interventions may be tried without clear benefit to the patient, including, but not limited to, supplemental oxygen. Therefore, it is important to consider alternative, albeit rare, diagnoses, including hemoglobinopathies with abnormal oxygen binding properties. Mutations in the structure of alpha- and beta-globin chains can alter the affinity of hemoglobin for oxygen, and changes in oxygen affinity may result in changes in the oxygen saturation detected by pulse oximetry. These changes may or may not be of clinical significance. This case report describes Hemoglobin Sunshine Seth, a rare low-oxygen-affinity hemoglobin variant presenting as reduced peripheral oxygen saturation in an otherwise well-appearing infant male.

## 1. Introduction

Low peripheral oxygen saturation in newborns can be due to cardiac or pulmonary disease. In the absence of cardiopulmonary pathologic conditions, other causes, such as hemoglobinopathies associated with low oxygen affinity, should be considered. Normal adult hemoglobin (Hb) is composed of a tetramer of 2 *α*-globin and 2 *β*-globin chains. Genetic mutations in these chains can affect the affinity of Hb for oxygen. If Hb affinity for oxygen is increased, oxygen adheres tightly to Hb and oxygen delivery to peripheral tissues is impaired. More red blood cells are required to compensate for poor oxygen delivery, resulting in erythrocytosis. In contrast, if Hb affinity for oxygen is reduced, oxygen delivery to peripheral tissues increases [1]; this change may result in relative anemia, because fewer red blood cells are required for adequate oxygen delivery, and the patient may have cyanosis due to increased oxygen extraction by peripheral tissues [2].

Genetic mutations in Hb structure that alter its affinity for oxygen may or may not be clinically significant. Hb variants with high oxygen affinity, including Hb Chesapeake, Hb Montefiore, and Hb Crete, often result in erythrocytosis and no clinically significant downstream effects [3–8]. Variants with low oxygen affinity, including Hb Kansas, Hb Beth Israel, Hb Saint Mande, and Hb Sunshine Seth, may result in anemia, cyanosis, and low peripheral oxygen saturation [9–14]. Notably, although pulse oximetry shows low peripheral oxygen saturation, oxygen delivery to the peripheral tissues is actually increased; the oximeter is simply detecting the relatively low percentage of oxygenated Hb in the bloodstream in the setting of increased oxygen extraction by peripheral tissues. Often, no clinically significant downstream effects are present when Hb has low oxygen affinity, and no treatment of hypoxemia or mild anemia is required.

Diagnosis of oxygen affinity-altering genetic mutations in Hb first requires determination of the Hb-oxygen dissociation curve and P50 value, the oxygen tension at which Hb is 50% saturated [15]. If these appear abnormal, protein testing and globin DNA sequencing can help to establish the genetic mutation and diagnosis; these are necessary for future genetic counseling [15].

We present a case of persistently low peripheral oxygen saturation in a 17-month-old boy who underwent extensive evaluations for cardiopulmonary disease. The patient had been receiving supplemental oxygen therapy. We determined that the patient had a low-oxygen-affinity hemoglobinopathy (Hb Sunshine Seth), which explained his documented mild anemia and low peripheral oxygen saturation. Oxygen therapy was discontinued, and the patient continues to thrive. This case illustrates the need to consider evaluation for abnormal-oxygen-affinity hemoglobinopathy in children with hypoxemia but no cardiopulmonary pathologic conditions.

## 2. Case Presentation

### 2.1. History and Clinical Findings

A 17-month-old white boy was referred to our pediatric hematology clinic for evaluation of persistent low oxygen saturation without evidence of cardiac or pulmonary pathologic conditions. During pregnancy, the patient's mother had HELLP syndrome (hemolysis, elevated liver enzymes, and low platelet count), and she had an emergent late-preterm cesarean delivery. Newborn screening showed evidence of an abnormal Hb variant, which did not prompt immediate evaluation. After delivery, the patient required respiratory support with continuous positive airway pressure. Pulse oximetry showed that he had persistently low oxygen saturation (range, 80%–85%), and he was quickly transitioned to oxygen supplementation via low-flow nasal cannula (LFNC). He was discharged from the hospital and continued to receive 0.25 L/min oxygen via LFNC to maintain peripheral oxygen saturation greater than 93%. He required supplemental oxygen to maintain adequate peripheral saturation while awake and asleep.

Prior evaluation included computed tomography of the chest, flexible bronchoscopy, and multiple echocardiographic examinations, all of which showed normal findings. When the patient was nearly 1 year old, Hb electrophoresis showed an *α*-globin chain abnormality, 16.1% abnormal Hb (reference value, 0.0%), 2.0% HbA_2_ (reference range, 2.0%–3.3%), and 0% fetal Hb (reference range, 0.0%–10.5%). The electrophoresis findings were thought to be unrelated to the patient's persistent hypoxemia. In addition, the patient had mild normocytic anemia. Laboratory evaluation demonstrated Hb 9.8 g/dL (mean (2 SD) reference value, 12 (10.5) g/dL) and mean corpuscular volume 86.1 fL (mean (2 SD) reference value, 78 (70) fL) [16, 17].

### 2.2. Clinical Course and Diagnosis

Because of the diagnostic uncertainty related to his care, the patient was referred to our institution for additional evaluation. The patient had no signs or symptoms suggestive of cardiopulmonary disease (e.g., cough, cyanosis, dyspnea, tachypnea, and wheezing), even when supplemental oxygen was suspended. His activity level was normal, and he had no history of recurrent respiratory infections. He was a well-appearing toddler. His body mass index was at the 36th percentile, and his weight was at the 23rd percentile. Physical examination showed no evidence of perioral or peripheral cyanosis, clubbing, or stigmata of chronic hypoxemia. We did not identify any heart murmurs, abnormal lung sounds, or other pertinent cardiopulmonary conditions. Evaluation of the family history did not identify individuals with a blood disorder, cyanosis, oxygen dependency, or other similar clinical presentations.

**Figure 1:**
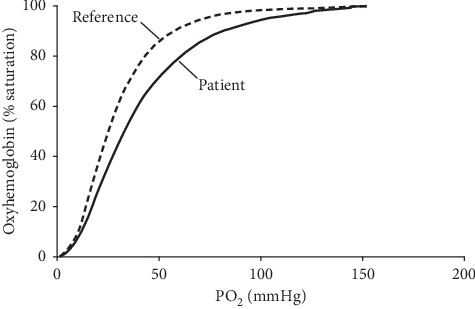
Hemoglobin-oxygen dissociation curve. The patient's curve is shifted to the right of the reference curve, consistent with reduced oxygen affinity. PO_2_ indicates partial pressure of oxygen.

On the basis of these findings, further investigations were performed to identify possible abnormal-oxygen-affinity hemoglobinopathies. A P50 oxygen-Hb assay showed a right-shifted Hb-oxygen dissociation curve (P50, 33 mm Hg; reference range, 24–30 mm Hg) (Figure 1). Hb electrophoresis testing with cation-exchange high-performance liquid chromatography, capillary electrophoresis, and isoelectric focusing detected an abnormal variant peak (80.5% HbA, 2.2% HbA_2_, 1.4% fetal Hb, and 15.9% Hb variant). DNA sequencing confirmed a heterozygous GAC > CAC, Asp > His missense mutation at codon 94 in the *α*-globin gene (Human Genome Variation Society nomenclature, *HBA2* c.283 G > C, p.D95H), resulting in Hb Sunshine Seth, an *α*-globin variant with low oxygen affinity. The Hb variant percentage was slightly lower than expected (15.9%; reference range, 26.3%–28.3%) [18]; however, the patient had concomitant iron deficiency (ferritin, 16 mcg/L; reference range, 24–336 mcg/L), which may have decreased the variant percentage. His parents were not tested for the genetic mutation.

### 2.3. Management and Outcome

The patient's diagnosis was Hb Sunshine Seth, an *α*-chain Hb variant with low oxygen affinity, resulting in reduced peripheral oxygen saturation without underlying cardiac or pulmonary pathologic conditions. For our patient, identification of this Hb variant with low oxygen affinity allowed for the cessation of diagnostic testing and discontinuation of unnecessary supplemental oxygen treatment. The patient is doing well without supplemental oxygen therapy.

## 3. Discussion

We described a case of low peripheral oxygen saturation in a child who received a diagnosis of oxygen dependency without a cardiopulmonary abnormality to explain his supplemental oxygen requirement. Our patient had a heterozygous genetic mutation for Hb Sunshine Seth, a Hb variant with low oxygen affinity that results from a mutation in the *α*-globin chain (histidine is substituted for an aspartic acid residue at position 94). This substitution is located in the *α*_1_-*β*_2_ contact site, which participates in the conversion of Hb from the oxygenated, relaxed state to the deoxygenated, taut state. Three of the 5 reported *α*-globin variants with amino acid substitutions at this position (Asp > Asn in Hb Titusville, Asp > Ala in Hb Bassett, and Asp > His in Hb Sunshine Seth) have decreased oxygen affinity and are associated with anemia, cyanosis, and decreased peripheral oxygen saturation [19].

Our patient did not have arterial blood gas studies performed. Therefore, the exact percentage of his Hb saturated by oxygen in the arterial blood (SaO_2_) is not known. It is noted in the literature that some low-oxygen-affinity Hb variants will be characterized by concordant reduction in SaO_2_ and SpO_2_ (the oxygen saturation detected by pulse oximetry), while others will be characterized by discrepant values (reduced SpO_2_ but normal SaO_2_). There are no reported values of SaO_2_ for patients with Hb Sunshine Seth in the literature, and it is challenging to predict if these values would be concordant or discrepant [20, 21]. In either case, there is no true tissue hypoxia. The pulse oximeter is simply detecting the relatively reduced ratio of oxygenated to deoxygenated Hb in the bloodstream, in the setting of increased oxygen extraction by peripheral tissues, resulting in a low peripheral SpO_2_ measurement.

The sigmoidal Hb-oxygen dissociation curve emphasizes 2 characteristics of Hb-oxygen binding. The first is cooperativity, a phenomenon that improves oxygen affinity in high-oxygen environments (e.g., the lungs). When Hb is partially saturated by oxygen, the protein is in a relaxed state, and affinity for oxygen in the remaining binding sites is increased; therefore, Hb binds slowest to the first oxygen molecule, and the speed of binding increases for each subsequent oxygen molecule. This cooperativity accounts for variations in the slope and the sigmoidal shape of the dissociation curve. The second defining characteristic is the modifiable oxygen affinity, which can change in response to temperature, pH (acidosis vs. alkalosis), chemical binding of alternative compounds (e.g., carbon dioxide), and the presence of 2,3-bisphosphoglycerate (the major modulator of Hb-oxygen affinity in humans). These modifiers can increase or decrease affinity for oxygen and shift the dissociation curve along the *x*-axis [1].

Hb Sunshine Seth is a rare low-oxygen-affinity Hb variant. It has been sequenced only 11 times in more than 20 years at our institution, and an additional 10 to 15 cases have been reported in the literature [22–24]. All reported infants appeared normal or slightly cyanotic at birth, and pulse oximetry showed low peripheral oxygen saturation (range, 76%–84%) [18]. The genetic mutation has been detected with cord blood screening, maternal screening, or evaluation of known familial cases. The mutation has not been associated with obvious deleterious effects on the hematologic parameters or health of any described patient, and treatment, including oxygen supplementation, for anemia, and/or special precautions, has not been required [22–24]. Importantly, these points must be emphasized to patients, family members, and other health care providers, because unnecessary and costly evaluations and interventions for hypoxemia are common.

In summary, genetic mutations in Hb resulting in altered oxygen affinity are rare but important considerations for patients with unexplained erythrocytosis (associated with high-oxygen-affinity Hb variants) or unexplained anemia, cyanosis, and low peripheral oxygen saturation (associated with low-oxygen-affinity Hb variants). Early identification of these variants may prevent unnecessary and invasive diagnostic testing, such as cardiac and pulmonary studies, for patients with unexplained hypoxemia.

## Abbreviations

Hb: Hemoglobin
LFNC: Low-flow nasal cannula.
